# Systematic Assessment of Visible-Light-Driven Microspherical V_2_O_5_ Photocatalyst for the Removal of Hazardous Organosulfur Compounds from Diesel

**DOI:** 10.3390/nano11112908

**Published:** 2021-10-30

**Authors:** Iqrash Shafiq, Murid Hussain, Sumeer Shafique, Parveen Akhter, Ashfaq Ahmed, Raja Shahid Ashraf, Moonis Ali Khan, Byong-Hun Jeon, Young-Kwon Park

**Affiliations:** 1 Department of Chemical Engineering, COMSATS University Islamabad, Lahore Campus, Defence Road, Off Raiwind Road, Lahore 54000, Pakistan; iqrash.shafiq@gmail.com (I.S.); sumeer.shafiq2013@gmail.com (S.S.); drahmed85@uos.ac.kr (A.A.); 2 Refinery Division, Pak-Arab Refinery Limited “Company” (PARCO), Corporate Headquarters, Korangi Creek Road, Karachi 12345, Pakistan; 3 Department of Chemistry, The University of Lahore, 1-km Defence Road, Off Raiwind Road, Lahore 54000, Pakistan; parveen.akhter@chem.uol.edu.pk; 4 School of Environmental Engineering, University of Seoul, Seoul 02504, Korea; 5 Department of Chemistry, Government College University, Lahore 54000, Pakistan; rajashahid@gcu.edu.pk; 6 Department of Chemistry, College of Science, King Saud University, Riyadh 11451, Saudi Arabia; mokhan@ksu.edu.sa; 7 Department of Earth Resources and Environmental Engineering, Hanyang University, Seoul 04763, Korea; bhjeon@hanyang.ac.kr

**Keywords:** V_2_O_5_ microspheres, visible-light activity, diesel oil, straight-run diesel, photocatalytic oxidative desulfurization

## Abstract

The organosulfur compounds present in liquid fuels are hazardous for health, asset, and the environment. The photocatalytic desulfurization technique works at ordinary conditions and removes the requirement of hydrogen, as it is an expensive gas, highly explosive, with a broader flammability range and is declared the most hazardous gas within a petroleum refinery, with respect to flammability. The projected work is based on the synthesis of V_2_O_5_ microspheres for photocatalytic oxidation for the straight-run diesel (SRD) and diesel oil blend (DOB). The physicochemical properties of V_2_O_5_ microspheres were examined by FT-IR, Raman, UV-vis DRS, SEM, and Photoluminescence evaluations. The as-synthesized photocatalyst presented a trivial unit size, a narrow bandgap, appropriate light-capturing capability, and sufficient active sites. The desulfurization study discovered that the anticipated technique is substantial in desulfurizing DOB up to 37% in 180 min using methanol as an interfacing agent. Furthermore, the outcome of employing a range of polar interfacing solvents was examined, and the 2-ethoxyethanol elevated the desulfurization degree up to 51.3%. However, the anticipated technology is constrained for its application in sulfur removal from SRD. Additionally, the mechanism for a photocatalytic reaction was seen in strong agreement with pseudo-first-order kinetics. The investigated photocatalyst exhibited a compromised recyclability and regeneration tendency.

## 1. Introduction

There is a high demand for liquid fuels globally. The organosulfur compounds present in liquid fuels are poisonous for the petroleum refinery’s reforming catalyst and cause a negative influence on the ambient air quality by the release of the oxides of sulfur formed during the combustion process, leading towards the increase in global warming, haziness, acidic raining, and to becoming a significant source of air pollution. Furthermore, the existence of SO_x_ in the atmosphere is very hazardous, having an adverse effect on the climate and becoming a source of severe health problems such as respiratory disorder, asthma, heart issues, and liver damage [1,2,3].

The shift towards cleaner fuels has received urgent attention to ensure the preservation of the environment. Legislative authorities have structured stringent sulfur requirements for this purpose [4]. The developed countries such as Japan have introduced the maximum sulfur limit of ten ppm in motor gasoline since 2017, regulated by JIS K standard 2022. Similarly, the US has controlled the sulfur limit of ten ppm in diesel oil since 2017. Likewise, China has limited the sulfur contents of gasoline and diesel to ten ppm as per V-generation fuels [5]. 

Numerous technologies have been presented to meet the standard requirements regarding cleaner fuels [3]. These technologies include oxidative desulfurization [6], adsorptive desulfurization [7], electrochemical and biological desulfurization [8], hydrodesulfurization (HDS) [9,10], and employment of photocatalyst for the enhancement of oxidative desulfurization [11] in the production of cleaner fuels. All the conventional and even the latest refining units opted for the HDS technology for producing cleaner refinery products. The progression of HDS technology demands stringent process requirements such as high temperatures and pressures, larger complex reactors, dense catalyst loadings requiring high volumes, elevated capital and operational costs, expensive catalysts, and expensive gas, i.e., hydrogen requirements [3]. Moreover, the technology faces many challenges in the removal of refractory sulfur compounds owing to the compromised hydrodesulfurization reactivities of these stringent sulfides [4,12,13,14,15]. 

On the other hand, several non-HDS technologies have been introduced globally, including oxidative-desulfurization (ODS), adsorptive-desulfurization (ADS), and bio-desulfurization (BDS), among others [5]. The refractory organosulfur compounds have high reactivities in the oxidation systems compared with that of hydrogenation ones, making the oxidative desulfurization technology a superior technique for the deep desulfurization of liquid fuels [2]. Moreover, the ease of proceeding, inexpensive oxidants, and the system’s operability at ambient temperature and pressure make the system hard to match.

Generally, hydrogen peroxide is used as an oxidant for oxidative desulfurization because of its high oxidation ability [16]. However, the hazardousness, the cost, explosivity, transportation issues, safety reservations, and the probability of undesired reactions make it unsuitable as an effective oxidant for this system [2,17,18]. Several other oxidizing agents, including molecular oxygen, ozone, and tert-butyl hydroperoxide, are investigated for oxidative desulfurization systems [4]. Currently, the photons carried by the light irradiations have been examined for the initiation of the molecular oxygen as an oxidant for the removal of refractory sulfur compounds [3]. Henceforth, the technology of photocatalytic oxidative desulfurization has emerged with a significant potential for inexpensive, environment-friendly, and hydrogen-free smooth operability at ambient conditions [19,20]. 

Developing a photocatalyst with superior physicochemical characteristics is a challenge. Many photocatalytic materials have been reported for photocatalytic desulfurization applications, including ZnO, TiO_2_, CeO_2_, and WO_3_. Nevertheless, the activity of these materials upon a limited range of light irradiations affects their broader photocatalytic applicability [21,22,23,24]. Recently, vanadium pentoxide (V_2_O_5_) has received significant attention as a visible-light-driven photocatalytic material, owing to its slim bandgap energy of 2.40 eV, lower cost, and the two-dimensional layered structure [25]. Numerous routes have been developed to synthesize the V_2_O_5_ microstructure. Wang et al. reported mono-disperse V_2_O_5_ microspheres with vanadium isopropoxide using the sol–gel method by controlled annealing method for Li^+^ ion batteries [26]. Yin et al. synthesized porous V_2_O_5_ micro and nanotubes by chemical vapor CVD technique for RhB degradation [27]. Xue et al. synthesized porous V_2_O_5_ micro and nanotubes by chemical vapor CVD technique for RhB degradation [28]. Gotić et al. produced V_2_O_5_ powder using the sol–gel approach [29], Uchaker et al. synthesized the porous V_2_O_5_ microspheres by polyol solvothermal process [30] for a range of photocatalytic applications. However, to the best of our knowledge, so far, neither the V_2_O_5_ photocatalytic semiconductor materials have been investigated for the deep desulfurization of petroleum refinery end-products, nor has any research work been conducted to investigate the potential of photocatalytic desulfurization technology for the sulfur-rich streams such as straight-run diesel.

Herein, the V_2_O_5_ photocatalyst having a yolk-shell micro-spherical structure has been developed for sulfur removal application by using a hydrothermal approach. The as-synthesized photocatalysts exhibited adequate yield, inexpensive route, and a simple synthesis procedure at controlled reaction conditions. The visible-light-driven photocatalytic desulfurization activity of micro-spherical V_2_O_5_ has been examined. The as-synthesized V_2_O_5_ microspheres revealed moderate desulfurization activity performance for the diesel oil blend and the fairly compromised organosulfur compounds conversion for the straight-run diesel.

## 2. Experimental Methods 

### 2.1. Materials

The materials employed for the synthesis of as-synthesized photocatalyst include ammonium metavanadate (NH_4_VO_3_, 99.0%, Sigma-Aldrich, St. Louis, MO, USA), ethanol (C_2_H_5_OH, 99.99%, Merck chemicals, Darmstadt, Germany), sodium hydroxide (NaOH, 98.9%, Fisher scientific, Richardson, TX, USA), hydrochloric acid (HCl, 37%, CARLO ERBA Reagents, Barcelona, Spain), N,N-dimethylformamide (HCON(CH_3_)_2_, 99.8%, Sigma-Aldrich, St. Louis, MO, USA), 2-ethoxyethanol (C_2_H_5_OCH_2_CH_2_OH, 99%, Sigma-Aldrich, St. Louis, MO, USA), acetonitrile (CH_3_CN, 99.8% purity, Merck chemicals, Darmstadt, Germany), β-butyrolactone (C_4_H_6_O_2_, 98%, Merck chemicals, Darmstadt, Germany), and methanol (CH_3_OH, 99.99%, Merck chemicals, Darmstadt, Germany). Deionized water was used as a synthesis and cleaning reagent throughout the experimentation. 

### 2.2. Synthesis of Microspherical V_2_O_5_

V_2_O_5_ microstructures were synthesized by using a hydrothermal synthesis approach. In a typical synthesis, a stoichiometric amount of NH_4_VO_3_ was dispersed in the demineralized water by stirring for four hours. The attained solution was gently poured into the Teflon-lined stainless-steel autoclave and heated overnight to obtain a yellowish solution. A stoichiometric amount of ethanol was introduced into the solution, stirred for 30 min and placed for aging at room temperature for an hour, resulting in the immediate formation of yellowish precipitates. The precipitates were separated through centrifugation and dried at 80 °C, followed by calcination at 400 °C for four hours to yield pure micro-spherical vanadium oxide crystals (V_2_O_5_). 

### 2.3. Sample Characterization

The details regarding chemical bonds and the presence of functional groups were investigated by FT-IR (Nicolet 20 SX FT-IR Spectrophotometer, Thermo Scientific, Waltham, MA, USA) between the scan series from 3500 to 500 cm^−1^. The textural examination was carried out by SEM (Philips XL 30 FED SEM, Philips Research, Cambridge, MA, USA). Raman investigation and fluorescence measurements were taken by Raman InVia Raman Microscope instrument, Renishaw plc, Kingswood, Wotton-under-Edge, UK. The machine was equipped with an argon laser ion excitation at 514 nm wavelength. The samples were scanned between 100–1000 cm^−1^ range for 10 s. Bandgap measurements were taken by UV-Vis Diffuse Reflectance Spectroscopy (JASCO V-770 equipment). Moreover, sulfur content identification was carried out on an X-ray wavelength dispersive sulfur analyzer (ASW-2, Bourevestnik, JSC, St Petersburg, Russia). 

### 2.4. Photocatalytic Studies

The diesel oil blend and straight-run diesel containing 410 and 12,100 ppm sulfur contents, respectively, were investigated for the aerobic oxidative desulfurization capability under visible light irradiations. The samples were taken from a petroleum refinery and given the tags of DOB-0.04 and SRD-1.21, based on their sulfur contents in weight percentages. The reactions were performed in a jacketed beaker provided with tap-water circulation, placed on the magnetic stirrer with heating provision. The reaction solution was irradiated with a 500 W xenon lamp. N,N-dimethylformamide, 2-ethoxyethanol, acetonitrile, β-butyrolactone, and methanol were used as a polar solvent for the provision of the oxidation reaction media as well as for the oxidation product separation. The reaction solution was aged in darkness to achieve the adsorption–desorption equilibrium among the organosulfur compounds and the photocatalytic powder. Frequently, 4–8 mL reaction solution was separated and passed through a 0.2 μm syringe membrane to determine the amount of the sulfur contents in the solution. 

## 3. Results and Discussion

### 3.1. Characterization of the Photocatalysts

**Figure 1:**
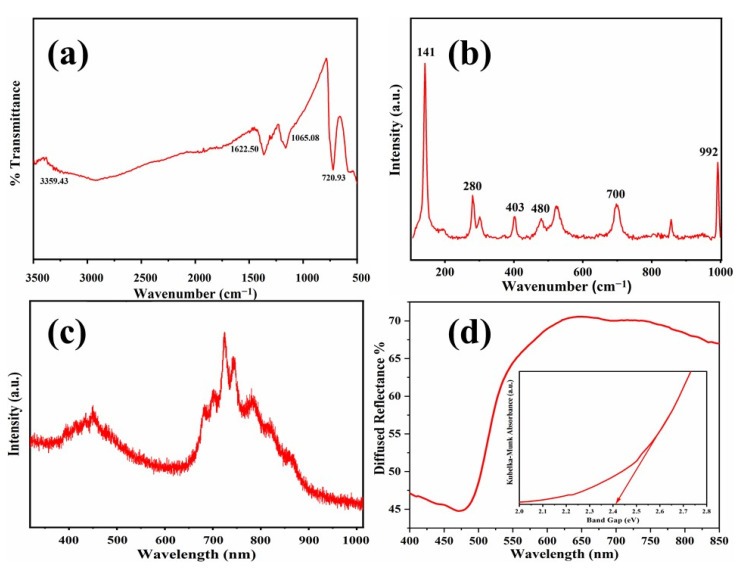
FT-IR (**a**), Raman (**b**), PL (**c**) and UV-Vis DRS (**d**) spectrum for micro-spherical V_2_O_5_ photocatalyst.

FT-IR study was carried out to investigate bond analysis and chemical composition of V_2_O_5,_ as shown in Figure 1a. The solid IR absorbance bands of hydrated V_2_O_5_ located at about 3359.43 and 1622.50 cm^−1^ are dispensed to O–H stretching as well as bending vibrations, respectively. Probably such space broadening by the introduction of primary H_2_O structure may be presented for insertion of larger cations [31]. The bands from 500 to 800 cm^−1^ are ascribed to symmetric as well as asymmetric modes of vibration of V–O, and the bands from 800 to 1020 cm^−1^ are correlated to V=O vibrations in V_2_O_5_ [32]. The distinctive bands at 1065.08 and 720.93 cm^−1^ are allotted to terminal oxygen stretching in V=O and asymmetric stretching vibrations of the V–O–V bridge, respectively [33].

The Raman spectroscopy was carried out in a wavelength range from 100 to 1000 cm^−1^ for V_2_O_5,_ as shown in Figure 1b. The prevailing band at 141 cm^−1^ was related to V–O–V vibrations, and its presence reveals the microstructure of V_2_O_5_. The band that appeared at 992 cm^−1^ communicates to the stretching mode of terminal oxygen (V^+5^=O), which is related to the crystallinity of structure [34]. The band at 700 cm^−1^ is allotted to the stretching mode of doubly coordinated oxygen (V_2_–O) due to corner shared oxygen. The small band at 480 cm^−1^ is allotted to V–O–V bridging and V_3_–O bonds in a triply coordinated oxygen system [35]. The prominent bands positioned at 403 and 280 cm^−1^ are delegated to the bending vibrations of V–O bonds [36].

Figure 1c reveals the PL spectra of the V_2_O_5_ nanostructure. The PL band shows two peaks positioned around 400–480 nm and 650–850 nm. The peak around 400–480 nm is initiated by the transition from the top of the conduction band to the valance band. The emission band at 650–850 nm is an extrinsic transition produced by oxygen vacancies due to the calcination process. The PL spectrum intensifies due to the reorganization of the structure when calcination at elevated temperature [37].

The diffuse reflectance spectrum (DRS) of as-synthesized V_2_O_5_ is shown in Figure 1d. Pure V_2_O_5_ shows broad absorption in the visible light region [38] and displays a strong absorption band at 500 nm [36]. The peak at the beginning of the visible spectrum at about 400 to 500 nm suggests that as-synthesized V_2_O_5_ can be used as an efficient photocatalyst concealing the visible range [39]. The optical bandgap of V_2_O_5_ was intended to be 2.41 eV using Tauc’s plot presented in the inset of Figure 1d.

**Figure 2:**
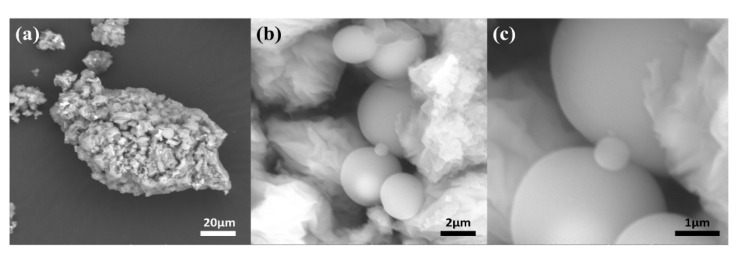
SEM micrographs of micro-spherical V_2_O_5_ photocatalyst at (**a**) low, (**b**) medium and (**c**) high resolution.

The structural details and morphology of as-synthesized sample V_2_O_5_ were examined by SEM, as shown in Figure 2. The figure reveals smooth microspheres having a well-disposed, nonagglomerated structure, demonstrating that a product with a high yield can be obtained using the described hydrothermal synthesis method. The magnified images of SEM (Figure 2b,c) indicate that the microspheres have a yolk-shell structure. Figure 2c reveals that the microspheres of V_2_O_5_ are uniform and perfectly maintained. There is no noticeable shrinkage or structural defects in microspheres, which proposes outstanding structural strength.

### 3.2. Photocatalytic Activity

**Figure 3:**
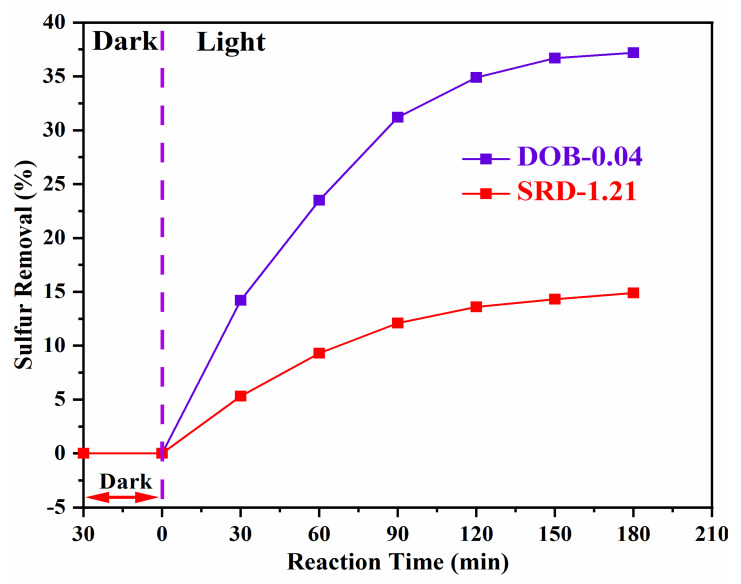
Photocatalytic desulfurization efficiency of V_2_O_5_ photocatalyst for diesel oil using methanol as an interfacing agent.

The photocatalytic desulfurization reactions were performed under visible-light illumination using as-synthesized photocatalytic material for 180 min with methanol as a solvent, as shown in Figure 3. Photocatalyst powder quantity of 3 g/L was added to the beaker containing the real diesel, 100 mL/min air flow rate was controlled, 20 °C temperature was achieved using the tap-water circulation, pH 4 was achieved by adding HCl and NaOH dropwise, and the reaction proceeded with moderate stirring. 

Figure 3 illustrates the photocatalytic sulfur removal ability of real diesel streams over a photocatalyst. No sulfur removal was observed during the first 30 min of stirring without visible-light irradiations, as no photogenerated charge carriers were available and no adsorbent characteristic material was employed. 

Upon visible-light irradiations, the photogenerated charges were generated at the surface of the photocatalyst resulting in the desulfurization of the intended streams by the adsorption of the sulfone in the polar solvent. The straight-run diesel, which typically contains up to 1 wt% sulfur contents and higher, generally contains a variety of sulfur species. The SRD-1.21 exhibited a significant desulfurization rate over the visible-light photocatalyst, reaching up to 14.93% within 180 min. On the other hand, the diesel oil blend, a mixture of desulfurized diesel streams received from different process units of a petroleum refinery, generally contains a significant fraction of refractory organosulfur compounds such as 4,6-DMDBT, which are known to be extremely reactive in the ODS system. The DOB-0.04 exhibited a desulfurization rate of 37.21% within 180 min.

The straight-run diesel is comprised of a range of inhibiting agents such as nitrogenous compounds, oxygen-containing species, etc., while most of the impurities such as metal, nitrogen, and oxygen-containing compounds, are removed from the diesel oil blend. The presence of the complex mixture of impurities in the straight-run diesel leads to restricted desulfurization rates for various organosulfur compounds. Moreover, the presence of a higher amount of sulfur contents in the diesel streams has a significant effect on the opaqueness of the solution. The diesel with less than 10 ppm sulfur depicts a pure water-like transparent color, while the color of the diesel increases from yellowish green to brownish color with the increase in the sulfur contents. Likewise, the diesel stream containing up to 1 wt% sulfur content exhibits brownish color, increasing the opacity and restricting the light penetration, thereby suppressing the production of photogenerated charge carriers and resulting in the decline of the desulfurization rate. This is one of the prominent reasons for the extremely low desulfurization rate of straight-run diesel, in addition to the presence of a range of least reactive organo-sulfur compounds. 

The obtained desulfurization values of the investigated real diesel streams are unacceptable with respect to the latest statutory requirements. However, the value obtained for the diesel oil blend is relatively high for converting the Euro-II sulfur specification to the Euro-III sulfur specification. Therefore, these experimental results indicate that the APODS is not suitable for desulfurizing straight-run diesel streams. The technology, however, has significant potential and could be improved through material modifications and further investigated for use in the desulfurization of the diesel oil blend.

### 3.3. Effect of Several Interfacing Agents

**Figure 4:**
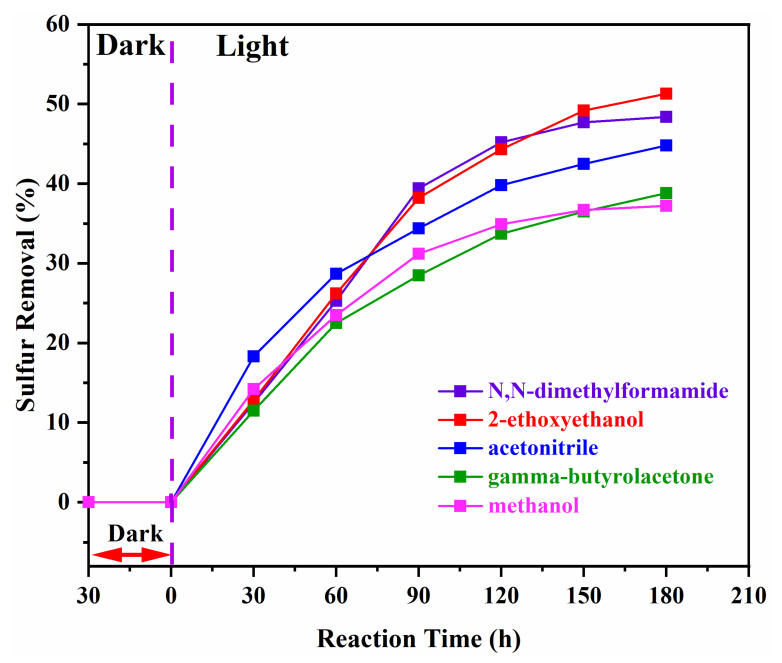
Effect of using (purple line) N,N-dimethylformamide, (red line) 2-ethoxyethanol, (blue line) acetonitrile, (green line) gamma-butyrolactone, and (pink line) methanol on photocatalytic desulfurization of diesel oil.

The effect of using different solvents on the photocatalytic oxidative desulfurization performance of diesel oil was also investigated. Different solvents such as N,N-dimethylformamide, 2-ethoxyethanol, acetonitrile, β-butyrolactone and methanol were used as a polar solvent for the provision of the oxidation reaction media as well as for the oxidation product separation. The total sulfur removal values varied by the nature of the solvent used. The highest desulfurization performance was obtained using 2-ethoxyethanol, and the lowest desulfurization rate was recorded using methanol, as shown in Figure 4. The different solvents such as N,N-dimethylformamide, 2-ethoxyethanol, acetonitrile, β-butyrolactone and methanol exhibited a total sulfur removal of 48.4%, 51.3%, 44.8%, 38.8%, and 37.2%, respectively. The mechanism of the photocatalytic oxidative desulfurization reveals that initially, the organosulfur compounds are extracted into the polar phase, and therein oxidation occurs, resulting in the production of corresponding sulfoxides and sulfones [40]. It is worth mentioning that the polar solvent plays a key role in the solvation of the organosulfur compounds along with the molecular oxygen. The withdrawal of the organosulfur compounds from the oily phase into the polar phase takes time and requires adequate agitation. The shifting of the organosulfur compounds is not the controlling step in the course of photocatalytic oxidative desulfurization. The polar solvent significantly facilitates the oxidative desulfurization reaction by providing an efficient pathway for the transfer of electrons present in the group of highly reactive species, contributing to the high desulfurization rates [41]. Summarily, the desulfurization rate using different solvents are found in following trend: 2-ethoxyethanol > N,N-dimethylformamide > acetonitrile > β-butyrolactone > methanol.

### 3.4. Kinetic Study

The real diesel samples were obtained from a petroleum refinery, ranging from mercaptans to refractory organosulfur compounds. The organosulfur compounds exhibit diverse electron densities and different values of oxidation reactivities. Nevertheless, a detailed understanding of the photocatalytic desulfurization activities for these natural fuels with a range of organosulfur compounds is mandatory in order to proceed towards the commercialization of an advanced desulfurization technology. Herein, an effort to investigate the kinetic mechanism for identifying the route of oxidation reactions is carried out, and the rate of reaction is determined. The modest correlation for the photocatalytic desulfurization performance is as follow:

ln C_t_ = −k_app_ t + ln C_o_ (1)

where C_o_ and C_t_ are the concentrations of the sulfides species initially, and at the time t, respectively, and k_app_ is the apparent pseudo-first-order reaction rate [42]. 

Equation (1) is integrated to give,

ln (C_o_/C_t_) = k_app_ t, (2)

**Figure 5:**
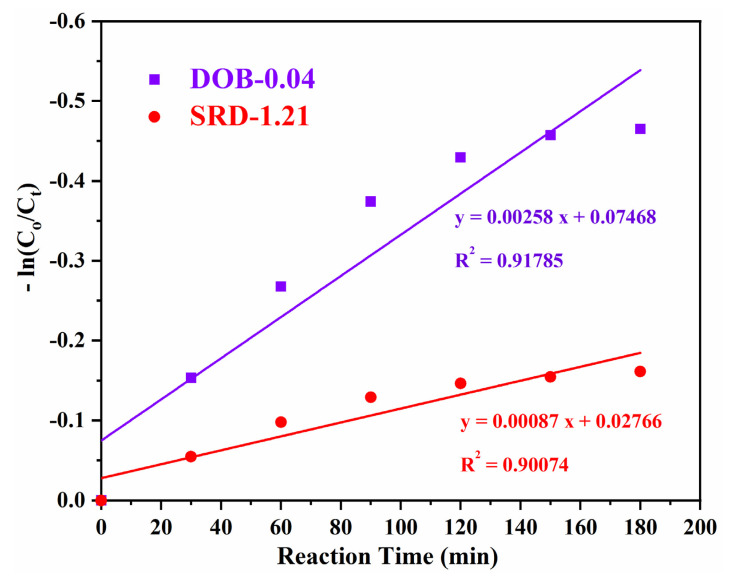
The pseudo-first-order kinetic fit for desulfurization diesel streams.

The desulfurization rates values obtained from the experiments are also investigated for their kinetic fit (Figure 5). The desulfurization reaction rate for the real diesel oil followed pseudo-first-order kinetics is revealed by the plot of −ln (C_o_/C_t_) against the time t. It is observed that all the obtained desulfurization rates for R^2^ surpass 0.9. The reaction rate coefficients (k_app_) values are obtained by measuring the slope of the straight line. The compatible k_app_ value for the DOB-0.04 and SRD-1.21 are 2.58 × 10^−3^ and 8.70 × 10^−4^ min^−1^, respectively. The k_app_ of DOB-0.04 is three times higher than SRD-1.21. These results suggest that the aerobic photocatalytic oxidation technology is sufficient for desulfurizing the diesel oil blend, while the system’s efficiency is not adequate to desulfurize the straight-run diesel effectively.

### 3.5. Proposed Mechanism

**Figure 6:**
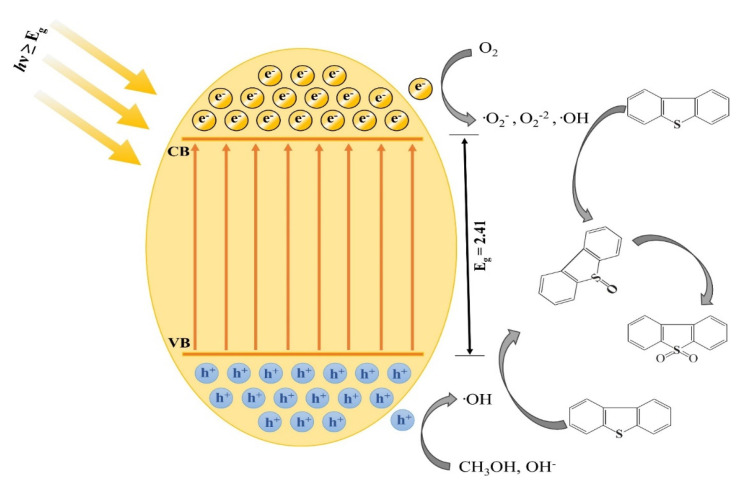
Proposed mechanism for photocatalytic desulfurization.

Figure 6 represents the visible-light-simulated generation of electron-hole pairs inside the V_2_O_5_ photocatalysts, and the comprehensive route for the photocatalytic desulfurization process is provided. The light photons absorbing on the photocatalyst surface, having energy greater than the bandgap to the semiconductor material, will excite the electrons from the valence band to the conduction band of the semiconductor and yield ·O_2_^−^ by reacting with the molecular oxygen [22]. The usage of air bubbling from the reaction solution provides an electron trap, preventing the recombination of electron-hole pairs [43].

The diffusion of the charged species at the 2/5 location of the thiophenic ring in the sulfides comprising of ring structures is generally the initiation step, forming a highly reactive oxidized species [44,45]. Afterward, the holes generated due to electrons excitation are mobilized towards the valence band and interact with OH^−^ producing ·OH. Conclusively, a sufficient number of intermediate species are generated near the photocatalyst to transform the sulfides (present closer to the oil-solvent interface) to eventually form sulfones. The charged oxidized species are soluble in the polar solvent and are removed from the reaction solution [46,47,48,49,50,51,52,53,54,55]. The possible mechanism is given by:

V_2_O_5_ + light photons → e^−^_(CB)_ + h^+^_(VB)_ (3)

H^+^_(VB)_ + OH^−^ _(ads)_ → ·OH (4)

e^−^_(CB)_ + O_2_ → ·O_2_^−^ (5)

·O_2_^−^ + sulfides → sulfoxides (6)

h^+^ + sulfoxides → sulfones (7)

### 3.6. Photostability Test

**Figure 7:**
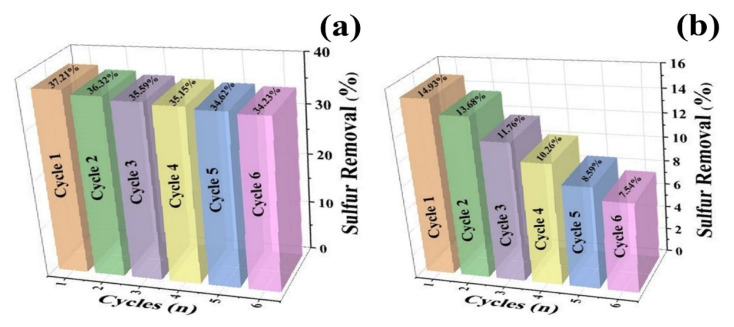
Recyclability of the micro-spherical V_2_O_5_ photocatalyst over desulfurization of (**a**) diesel oil blend and (**b**) straight-run diesel.

The deactivation of the catalyst material is the foremost challenge in the commercialization of any catalytic material. Figure 7 depicts the six successive cycles of reusability of the V_2_O_5_ photocatalytic material. The deactivation of the photocatalytic material was significant in both cases. i.e., (a) diesel oil blend and (b) straight-run diesel. The photocatalytic desulfurization of diesel oil blend revealed the desulfurization rate decline from 37.21% to 34.23% (Figure 7a), while the desulfurization rate of decline for straight-run diesel was even more significant, ranging from 14.93% to 7.54% (Figure 7b). The deactivation rate was found to be more dominant in the case of straight-run diesel due to the presence of a diverse range of impurities adhering on the active sites and leading towards proceeding the semiconductor material towards an inactive state. Therefore, it could be concluded that there is a requirement for robust photocatalytic materials necessary for the efficient desulfurization of the diesel oil blend and the straight-run diesel.

### 3.7. Structural Stability of Photocatalyst

**Figure 8:**
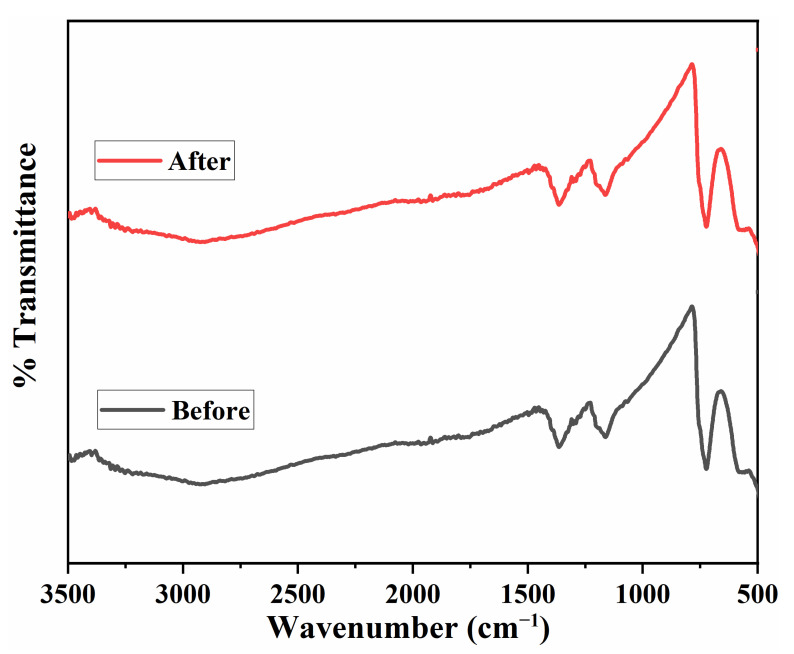
Structural stability test of the micro-spherical V_2_O_5_ photocatalyst.

The sample of the used photocatalyst was taken and washed thoroughly with deionized water and dried at 110 °C to remove the entrapped moisture altogether. The obtained material was again characterized with the Fourier Transmission Infrared Spectroscopy to obtain sufficient details regarding the functional group presence and the structural details. The obtained spectrum was compared with the FT-IR spectrum of the fresh catalyst, as shown in Figure 8. Upon comparison, no significant change in both FT-IR patterns was observed, revealing that the photocatalyst deactivation occurred due to some physical adhesions or other possible variations in the physical aspects, and no chemical change has taken place. Therefore, it could be deduced that the obtained spent photocatalyst could be regenerated by physical treatments and will be capable of being re-employed for photocatalytic desulfurization applications. 

## 4. Conclusions

The organosulfur compounds present in liquid fuels, and associated SO_x_ generation on combustion, are responsible for the deactivation of refinery reforming catalysts, generation of auto-ignitable pyrophoric sludges in refinery vessels, acidic rain, structural erosion, and premature deaths, and henceforth declared as hazardous for health, industry, and the environment. The hydrodesulfurization technique employed in the majority of the petroleum refineries is limited in the production of sulfur-free fuels due to the challenges encountered in the desulfurization of the refractory organosulfur compounds. The study is intended to investigate the photocatalytic desulfurization process capability for the desulfurization of the diesel oil blend and the straight-run diesel stream. A renewed semiconductor material with supreme physicochemical properties was selected, and the capability of photocatalytic desulfurization performance on real diesel streams was investigated. The rate of photocatalytic desulfurization of diesel oil blend was found higher than that of the straight-run diesel stream due to the less complex range of inhibiting agents and more light penetration due to less opacity and the presence of more reactive organosulfur compounds. The desulfurization rate for straight-run diesel was significantly lower, and the technology is not yet ready for the commercial desulfurization of straight-run diesel streams. However, the technology exhibited sufficient potential for the up-gradation of the diesel oil end-product grade by polishing the final blend. The investigation of the variance in the desulfurization rate using different solvents is found in the following trend: 2-ethoxyethanol > N,N-dimethylformamide > acetonitrile > β-butyrolactone > methanol. Furthermore, the kinetic study revealed a strong agreement with the pseudo-first-order kinetic fit.

## Data Availability

The data presented in this study are available on request from the corresponding author.
